# Emerging roles of gap junction proteins connexins in cancer metastasis, chemoresistance and clinical application

**DOI:** 10.1186/s12929-019-0497-x

**Published:** 2019-01-14

**Authors:** Jun-I Wu, Lu-Hai Wang

**Affiliations:** 1 Institute of Molecular and Genomic Medicine, National Health Research Institutes, Miaoli County, Taiwan; 2 Department of Life Sciences, National Central University, Taoyuan, Taiwan; 3 Graduate Institute of Integrated Medicine, China Medical University, Taichung, Taiwan; 4 Chinese Medical Research Center, China Medical University, Taichung, Taiwan

**Keywords:** Connexins, GJIC, Metastasis, Chemoresistance, Therapeutics

## Abstract

Connexin, a four-pass transmembrane protein, contributes to assembly of gap junctions among neighboring cells and thus facilitates gap junctional intercellular communication (GJIC). Traditionally, the roles of connexins were thought to mediate formation of hemichannels and GJIC assembly for transportation of ions and small molecules. Many studies have observed loss of GJIC, due to reduced expression or altered cytoplasmic localization of connexins, in primary tumor cells. Connexins are generally considered tumor-suppressive. However, recent studies of clinical samples suggested a different role of connexins in that expression levels and membrane localization of connexins, including Connexin 43 (Cx43, GJA1) and Connexin 26 (Cx26, GJB2), were found to be enhanced in metastatic lesions of cancer patients. Cx43- and Cx26-mediated GJIC was found to promote cancer cell migration and adhesion to the pulmonary endothelium. Regulatory circuits involved in the induction of connexins and their functional effects have also been reported in various types of cancer. Connexins expressed in stromal cells were correlated with metastasis and were implicated in regulating metastatic behaviors of cancer cells. Recent studies have revealed that connexins can contribute to cellular phenotypes via multiple ways, namely 1) GJIC, 2) C-terminal tail-mediated signaling, and 3) cell-cell adhesion during gap junction formation. Both expression levels and the subcellular localization could participate determining the functional roles of connexins in cancer. Compounds targeting connexins were thus tested as potential therapeutics intervening metastasis or chemoresistance. This review focuses on the recent findings in the correlation between the expression of connexins and patients’ prognosis, their roles in metastasis and chemoresistance, as well as the implications and concerns of using connexin-targeting drugs as anti-metastatic therapeutics. Overall, connexins may serve as biomarkers for cancer prognosis and as therapeutic targets for intervening metastasis and chemoresistance.

## Background

Cancer is the second leading cause of mortality worldwide [1]. While early-diagnosed cancer patients are possible for curative surgery and favorable long-term survival, patients diagnosed with metastasis have much lower survival rates [2]. Hence, metastasis accounts for about 90% of cancer-related death [3]. Metastasis is a multi-step process termed invasion-metastasis cascade [4]. During metastasis, cancer cells need to escape from their primary sites, migrate and invade the basement membrane. After intravasation into the blood or lymphatic vessels, cancer cells must survive during circulation, attach to the endothelium, and transmigrate or extravasate into target organs. Disseminated tumor cells have to survive and outgrow at target organs, namely to successfully colonize, to form metastatic tumors [3].

**Fig. 1:**
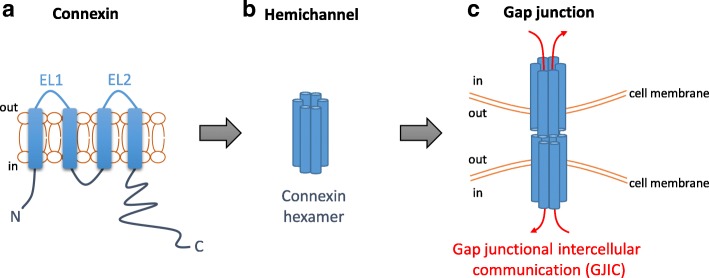
The assembly of connexins into gap junctions. **a** The topology of a gap junction protein (connexin). A connexin contains a cytoplasmic N-terminus (AT), four transmembrane domains, two extracellular loops (EL1 and EL2), a cytoplasmic loop, and a cytoplasmic C-terminal tail (CT). **b** Connexins assemble into hexamers to form a hemichannel. **c** Docking of two hemichannels from two neighboring cells forms a gap junction. The exchange of small (< 1 kDa) molecules mediated by gap junctions is called gap junctional intercellular communication (GJIC)

The family of connexins, or gap junction proteins, contains 21 members in human with shared structural features [5, 6]. They consist of one cytoplasmic N-terminus (AT), two extracellular loops (EL1 and EL2), one cytoplasmic loop, four transmembrane domains, and one C-terminal tail (CT) [5] (Fig. 1a). The amino acid sequences of EL1 and EL2 among connexin isotypes are highly conserved for hemichannels assembly [5]. In contrast, the sequences of the C-terminal tails of different connexin isotypes are highly variable [5]. Connexins can assemble into hexamers to form a hemichannel [6] (Fig. 1b). Two hemichannels from two neighboring cells form a gap junction (Fig. 1c), allowing transmission of small (< 1 kDa) molecules such as ions [7], metabolites [8, 9], and even anticancer drugs [10]. The gap junction-mediated exchange of materials among two neighboring cells is termed gap junctional intercellular communication (GJIC) (Fig. 1c).

Connexins may contribute to cellular phenotypes in multiple ways, namely, 1) GJIC, 2) C-terminal tail-mediated signaling, and 3) cell-cell adhesion during gap junction formation. Firstly, GJIC allows the direct exchange of ions and metabolites between neighboring cells [7–9]. For instance, Cx43-GJIC has been reported to promote the transmission of cAMP in MC3T3-E1 pre-osteoblasts [9]. Moreover, GJIC is generally lost in dividing cells [11, 12], which is critical to avoid transmission of metabolites among dividing cells and their non-dividing neighbors [13]. Cancer is a disease of dysregulated cell growth [14], and GJIC is frequently lost in primary tumor cells via reduced expression and/or cytoplasmic localization of connexins [15–19]. Overexpressed Cx43 mediated GJIC has been reported to inhibit tumor growth via facilitating transmission of cAMP [20], suggesting an inhibitory role of Cx43-GJIC in tumorigenesis. Secondly, connexins may also inhibit tumorigenesis via their C-terminal tails interacting with signaling mediators [21]. Thus, both cell surface Cx43 and cytoplasm-localized Cx43 (cytoplasmic Cx43) were considered as tumor suppressors.

This review focuses on the novel role of connexins in metastasis as revealed in recent studies. Cx43 and Cx26 displayed increased expression and membrane localization in metastatic lesions [18, 19]. Cx26-mediated GJIC was found to facilitate cancer cell detachment from one another via reducing cell adhesion molecules, leading to enhanced cancer cell migration as single cells [22]. On the other hand, Cx43-mediated GJIC was found to facilitate cancer cell adhesion to endothelial cells, leading to enhanced extravasation and metastasis [23–29]. The role of other connexins or their isotypes expressed in cancer stroma was reported in relatively fewer studies. In addition, cytoplasmic connexins-mediated signaling and connexins-mediated GJIC were found to regulate chemoresistance of cancer cells. Connexins are thus also capable of promoting cancer progression and suggested as potential biomarkers for cancer prognosis and as therapeutic targets against cancer metastasis and chemoresistance.

## Connexin 43

### The suppressing roles connexin 43 in tumorigenesis

Cx43 is one of the most studied connexin isotypes in cancer, and has been reported to play suppressive roles in tumorigenesis via GJIC or C-terminal tail-mediated signaling. Firstly, Cx43-GJIC was found to facilitate the transmission of cAMP, leading to increased p27 levels and reduced tumor growth [20]. Thus, Cx43-GJIC has been known to play an inhibitory role in tumorigenesis. Secondly, cell surface and cytoplasmic Cx43 may suppress tumor growth via their C-terminal tails that are capable of interacting with signaling mediators. The C-terminal tail (261–319) of Cx43 has been found to interact with β-catenin [30]. Cell surface Cx43 has been shown to colocalize with β-catenin at the contact areas of neighboring cells [31]. The binding of cell surface Cx43 and β-catenin reduced the amount of free β-catenin available for Wnt signaling, leading to regulation of cyclin D1 and anti-apoptotic Bcl-2, and reduced cell proliferation [31]. Similarly, the cytoplasmic Cx43 overexpressed in HT29 cells has been reported to co-immunoprecipitate with β-catenin and inhibit Wnt signaling [21]. Overexpression of cytoplasmic Cx43 in U251 and T98G glioma cells reduced the levels of anti-apoptotic Bcl-2 [32]. Overexpression of cytoplasmic Cx43 was found to reduce the proliferation of LH7 lung cancer cells and HT29 colon cancer cells [21, 33]. Thus, both cell surface and cytoplasmic Cx43 can play suppressive roles in cell growth via interacting with cytoplasmic signaling mediators.

The suppressing role of cell surface and cytoplasmic Cx43 was supported by studies of primary tumor tissues from cancer patients. In primary tumors, GJIC was generally found lost [15–17]. One possible way leading to loss of GJIC is by downregulation of connexin expression. For instance, lack of Cx43 expression was found in almost all primary melanoma tumors [34]. On the other hand, loss of GJIC could also be caused by cytoplasmic localization of connexins [18, 35]. In gastric cancer, Cx43 was only expressed in the cytoplasm of most primary tumor cells and was reversely correlated with lymph node metastasis [19]. Similar results were also reported in pancreatic cancer [36] and lung cancer [33, 37]. In certain tumors where Cx43 is detected at the cell surface, the levels of Cx43 were reversely correlated with lymph node metastasis and prognosis [38, 39] (Table 1). The above studies suggested the inhibitory roles of Cx43 in tumorigenesis.

**Table 1:** Cx43 expression in clinical samples and its correlation with patients’ clinical outcomes

Cancer Type	Clinical manifest	Ref.
Cx43 in primary tumor tissues		
Gastric	Cytoplasmic Cx43; reversely correlated with lymph node metastasis	[19]
Pancreatic	Cytoplasmic Cx43; reversely correlated with lymph node metastasis	[36]
NSCLC	Cytoplasmic Cx43; reversely correlated with lymph node metastasis	[33, 37]
Laryngeal cancer	Cell surface Cx43; reversely correlated with lymph node metastasis, 5-year overall survival, and recurrence	[38, 39]
Cx43 in metastatic lesions		
Breast	Cell surface Cx43; increased expression and membrane localization in lymph node metastases	[18]
Melanoma	Cx43 mRNA; elevated expression in metastatic tissues	[34]
Gastric	Cx43 mRNA; elevated expression in metastatic peritoneal tissues	[29]
Breast	Cx43 mRNA; elevated expression in metastatic tissues	[43–45]

*NSCLC* Non-small cell lung cancer

In addition, it is unclear the reason leading to the defect of Cx43 membrane trafficking in primary tumor cells. In myocardial cells, oxidative stress was found to inhibit the membrane trafficking of Cx43 [40]. While oxidative stress is known to be closely related to carcinogenesis [41, 42], factors leading to the defect of the Cx43 membrane trafficking in primary tumor cells are still unclear.

### Increased expression and membrane localization of connexin 43 in metastatic lesions

While examination of Cx43 levels in primary tumor tissues revealed a tumor-inhibitory role of Cx43, increased expression and membrane localization of Cx43 in metastatic lesions were reported in studies of multiple cancer types. In a study of breast cancer, the expression and membrane localization of Cx43 in metastatic lymph nodes were increased relative to their paired primary breast tumors [18]. In some cases, Cx43-positive metastatic lymph nodes were found in patients with Cx43-negative primary tumors [18]. Increased Cx43 mRNA levels were also found in metastatic tissues than their primary breast tumors [43–45]. Similar results were reported in studies of gastric cancer and melanoma [29, 34] (Table 1). The above studies suggested the potential involvement of cell surface Cx43 in metastasis.

### Connexin 43-mediated GJIC enhances cell-cell adhesion and extravasation

**Fig. 2:**
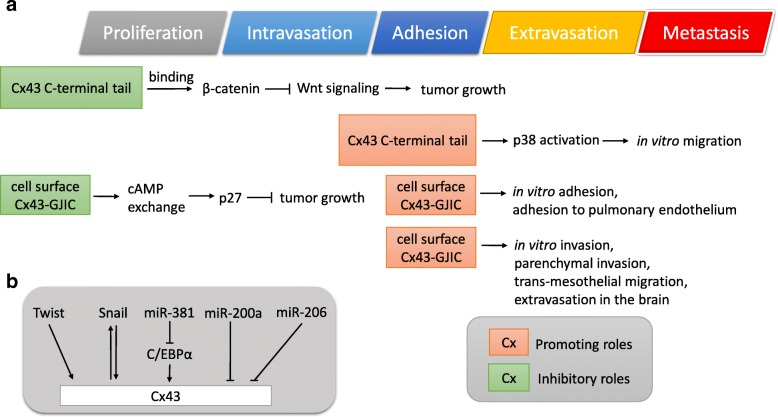
Functional roles and regulatory circuits of Cx43 in tumor progression. **a** The role of cytoplasmic Cx43-mediated effects or Cx43-GJIC in metastasis. **b** Transcription factors and microRNAs involved in the regulation of Cx43 expression

An important feature differentiating cell surface from cytoplasmic connexins is that cell surface connexins are possible for the formation of gap junctions. Besides facilitating the transmission of ions and metabolites, gap junction can also facilitate cell-cell adhesion [46, 47]. In a tail vein injection model, Cx43 was induced in the intra-tumor blood vessels and micro-metastatic foci at tumor cell-endothelial cell contact areas [23]. Moreover, functional GJIC was observed among melanoma and endothelial cells [48]. The Cx43-mediated GJIC was found to promote cell-cell adhesion. Overexpression of wild-type Cx43 enhanced the adhesion of 4T1 cells to the pulmonary endothelium, while decreased adhesion was observed in 4T1 cells overexpressing dominant-negative Cx43 mutant (Cx43-G138R) [23]. Similar results were found using a zebrafish model in that knockdown of Cx43 in 4T1 cells inhibited their extravasation in the brain and brain colonization [24]. In a chicken embryo metastasis model, treatment with gap junction inhibitor carbenoxolone (CBX) inhibited the brain metastasis of 4T1 cells injected into the main chorioallantoic membrane (CAM) vein of 14 days old chicken embryo [24]. Taken together, the above studies suggested that Cx43-GJIC promoted the adhesion of 4T1 cells to the endothelial cells, leading to enhanced extravasation and metastasis (Fig. 2a).

The promoting role of Cx43-GJIC in cell-cell adhesion and metastasis were also reported in prostate cancer, gastric cancer, and glioma cells. The PC-3 prostate cancer cells showed higher Cx43 levels and GJIC versus LNCaP prostate cancer cells [49]. Overexpression of Cx43 in LNCaP cells enhanced their GJIC, cell adhesion and invasion in vitro [25]. Moreover, in an intratibial injection mouse model, LNCaP cells overexpressing Cx43 showed elevated tumor incidence and osteolysis versus LNCaP cells expressing empty vector [25]. Conversely, knockdown of Cx43 in PC-3 cells inhibited wound healing migration and transwell invasion, while their proliferation abilities were unaffected [49]. In C6 glioma cells, overexpression of wild-type Cx43 promoted GJIC, cell-cell adhesion, invasion in vitro, and parenchymal invasion in vivo [26–28]. In contrast, overexpression of the C61S mutant of Cx43, which was unable to form gap junctions, did not enhance the cell-cell adhesion and parenchymal invasion [26]. In addition, the promoting effects of Cx43 in cell invasion were further improved when C6 cells were co-cultured with astrocytes [27]. In another study, Oliveria et al. showed that GJIC was required for the heterocellular coupling of glioma cells and astrocytes [28]. GL15 glioma cells expressing higher Cx43 levels displayed increased migration and heterocellular coupling with astrocytes than the lower expressing 8-MG glioma cells [28]. Treatment of GL15 cells with gap junction inhibitor CBX inhibited their heterocellular coupling with astrocytes and migration ability [28]. In a study of gastric cancer, wild-type Cx43 enhanced the diapedesis of BGC-823 and SGC-7901 gastric cancer cells, while the GJIC-defective mutant (T154A) did not [29]. Pre-treatment with gap junction inhibitor CBX inhibited the promoting effect of Cx43 on trans-mesothelial migration [29]. All these studies suggested that Cx43-GJIC enhanced cell-cell adhesion and extravasation during invasion and metastasis (Fig. 2a).

### Carboxyl-terminal tail of connexin 43 promotes cell migration via p38

Cx43, on the other hand, could also promote cancer migration in a GJIC-independent pathway. In the study by Behrens et al., expression of the C-terminal tail of Cx43 (CX43-CT) only did not increase the cell-cell coupling between HeLa cervical cancer cells, yet was sufficient to increase their p38 activity and migration [50]. Treatment with p38 inhibitor SB-203580 abolished the migration-promoting activity by Cx43-CT [50]. Similarly, knockdown of Cx43 abolished low dose γ-radiation-induced p38 activation, migration, and invasion in U87 and BMG-1 glioma cells [51]. The above studies suggested that Cx43 was inducible by low dose γ-radiation, leading to enhanced p38 activation and cell migration via Cx43 C-terminal tail (Fig. 2a).

Taken together, Cx43 may affect cancer behaviors via at least three different ways. Cx43-GJIC can facilitate not only the exchange of ions and metabolites but also cell-cell adhesion [46, 47]. Besides, the C-terminal tail of Cx43 interacting with various proteins may regulate signaling pathways such as Wnt signaling and p38, leading to reduced proliferation or enhanced migration [21, 50].

In addition, while GJIC is essential for the direct communication between neighboring cells, hemichannel is involved in the paracrine release of ions and metabolites [13]. So far there have been very few reports on the hemichannels in cancer cells. With that being said, indirect evidence has suggested the potential role of Cx43 hemichannels in cancer. Antibodies targeting the EL2 region of Cx43 have been found to inhibit Cx43 hemichannel but not GJIC [52]. Treatment with the monoclonal antibody targeting the Cx43-EL2 (MAbE2Cx43) decreased tumor burden and increased survival of BALB/c mice [53]. The above study suggested the potential role of Cx43 hemichannel in tumor growth. In 3T3 fibroblasts, Cx43 hemichannels have been found to promote the release of NAD+ and cyclic ADP-ribose, leading to increased proliferation [54]. Thus, it is speculated that Cx43 hemichannel in tumor cells may facilitate the release of NAD+, leading to enhanced tumor growth [55, 56], however, further investigation is needed.

### MicroRNAs that regulate metastatic behaviors of cancer cells via targeting connexin 43

Several microRNAs, including miR-200a, miR-206, and miR-381, were reported to regulate Cx43 expression in breast cancer cells. Cx43 in MDA-MB-231 cells was found to be targeted by miR-200a, which was decreased in metastatic breast cancer tissues [45]. Overexpression of Cx43 in MCF7 cells promoted their migration ability, which was abrogated by miR-200a [45]. In triple negative breast cancer MDA-MB-231 cells, Cx43 was found to be targeted by miR-206, resulting in the inhibition of cell adhesion [57]. Similar results were reported by another study that miR-206 inhibited the expression of Cx43, migration, and invasion in MCF-7 cells, while inhibition of miR-206 in MDA-MB-231 cells enhanced Cx43 expression, migration, and invasion [44]. In vascular smooth muscle cells, miR-206 induced by myocardin, the master regulator of smooth muscle gene expression [58], inhibited the expression of Cx43 [59]. In MDA-MB-231 cells, miR-381 indirectly inhibited Cx43 expression by targeting Cx43 promoter-binding transcription factor C/EBPα, leading to decreased cell migration [43] (Fig. 2b).

### The transcriptional regulation for connexin 43 expression

As described above, the expression of Cx43 was found to be increased in metastatic lesions [18, 19, 29, 34, 43–45, 60]. An important question would be what factors lead to the induction of Cx43 during metastasis. A number of studies revealed that Cx43 expression was transcriptionally regulated by Snail and Twist, which are known to be transcription factors activated at late stages of tumor progression [24]. Reciprocal regulation of Cx43 and Snail-1 expression was found in epithelioid prostate cancer cells [61]: overexpression of Snail enhanced Cx43 levels and trans-endothelial migration, whereas depletion of Cx43 inhibited Snail-1 expression and migration. In another study, expression of Twist, a key transcription factor regulating epithelial-mesenchymal transition (EMT) [62], enhanced Cx43 expression, extravasation and micro-tumor formation in the brain [24]. Knockdown of Twist reduced Cx43-mediated GJIC and micro-tumor formation [24]. The above studies suggested the involvement of Snail and Twist in the induction of Cx43 expression (Fig. 2b).

## Connexin 26

### Cytoplasmic connexin 26 is correlated with lymph node metastasis and poor prognosis

In primary tumor tissues of multiple cancer types, cytoplasmic Cx26 was found to be the predominant form of Cx26 [63–66]. Opposite to Cx43 described above, cytoplasmic Cx26 has been found to be correlated with tumor progression and poor prognosis. In a study of breast cancer, cytoplasmic Cx26 levels in breast tumor tissues were correlated with lymphatic vessel invasion and poor relapse-free survival [63]. In colorectal cancer, high cytoplasmic Cx26 levels in primary tumors were associated with venous invasion, lung metastasis and poor disease-free survival [64]. In follicular thyroid cancer, cytoplasmic Cx26 expression was also found to be associated with lymph node metastasis [65]. In papillary thyroid cancer, tumors with higher Cx26 levels showed a higher incidence of intra-glandular dissemination [65]. Likewise in esophageal squamous cell carcinoma (ESCC) tissues, high Cx26 levels were correlated with the high incidence of lymph node metastasis and poor patients’ survival [66]. High Cx26 mRNA levels in primary tumors were correlated with poor survival in melanoma and recurrence in breast cancer [24]. Together, the above studies suggested that cytoplasmic Cx26 in primary tumor cells may play a role in promoting metastasis (see Table 2).

**Table 2:** Cx26/Cx32 expression in clinical samples and their correlation with patients’ clinical outcomes

Cancer Type	Clinical manifest	Ref.
Cx26 in primary tumor tissues		
Breast	Cytoplasmic Cx26; associated with lymphatic vessel invasion and poor relapse-free survival	[63]
Colorectal	Cytoplasmic Cx26; associated with venous invasion, lung metastasis, and poor disease-free survival	[64]
FTC	Cytoplasmic Cx26; associated with lymph node metastasis	[65]
PTC	Cytoplasmic Cx26; associated with high incidence of intra-glandular dissemination	[65]
ESCC	Cytoplasmic Cx26; associated with lymph node metastasis and poor 5-year survival	[66]
Melanoma	Cx26 mRNA; associated with poor survival	[24]
Breast	Cx26 mRNA; associated with recurrence	[24]
Cx26 in metastatic lesions		
Breast	Cell surface Cx26; increased expression in lymph node metastases; cell surface Cx26 was only found in metastatic lesions	[18]
Colorectal	Cytoplasmic Cx26; increased expression in lung metastatic lesions	[64]
Cx32 in primary tumor tissues		
HCC	Cx32 mRNA; reversely correlated with histological grade and lymph node metastasis	[78]
	Cx32 mRNA; associated with low vascular invasion and high overall survival rate	[79]
Cx32 in metastatic lesions		
Breast	Cytoplasmic Cx32; increased expression in metastatic lymph nodes	[70]

*FTC* Follicular thyroid cancer, *PTC* Papillary thyroid cancer, *ESCC* Esophageal squamous cell carcinoma, *NSCLC* Non-small cell lung cancer, *HCC* Hepatocellular carcinoma

### Cytoplasmic connexin 26 promotes tumor growth, EMT, and invasion

**Fig. 3:**
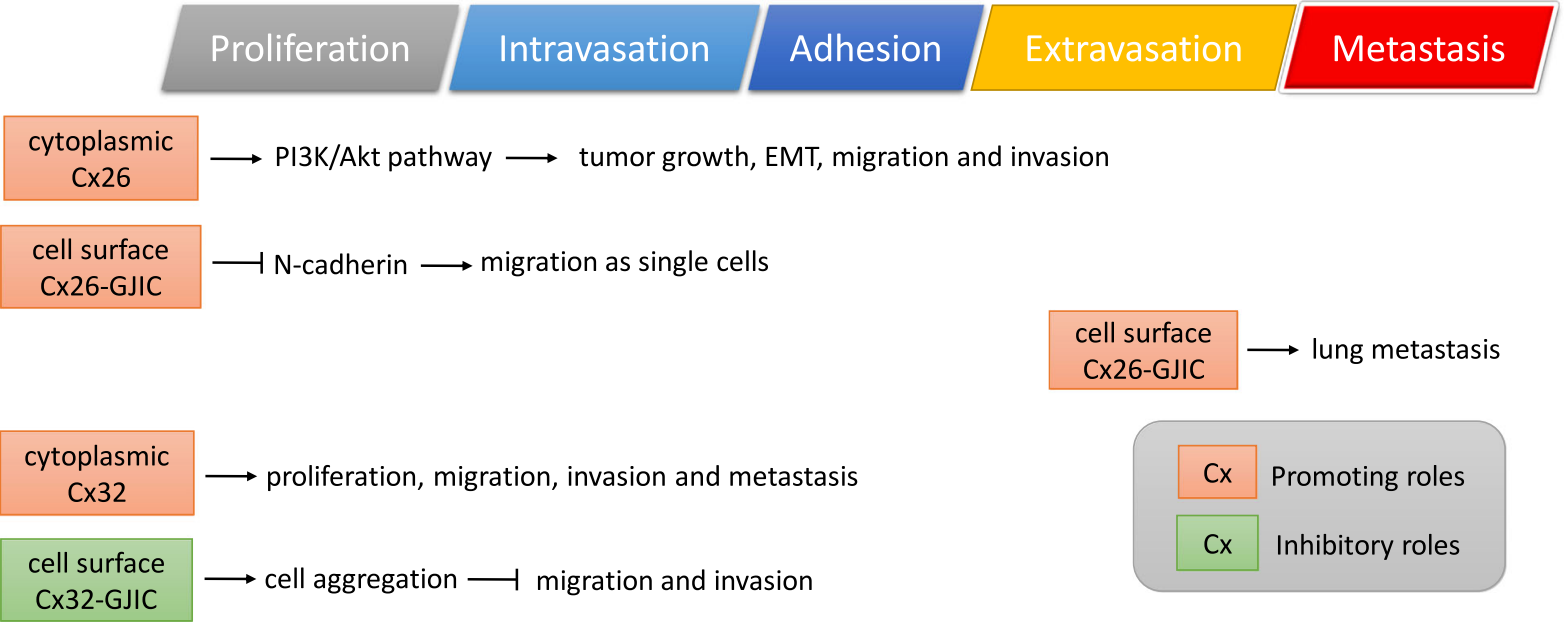
Functional roles of Cx26 and Cx32 in tumor progression

The role of cytoplasmic Cx26 was investigated in a study of NSCLC. Cx26 was found to be the predominant isotype of connexin in NSCLC cells [67]. In HCC827 and PC9 human lung adenocarcinoma cells, endogenous or overexpressed Cx26 was localized in the cytoplasm and thus cannot form functional GJIC [67]. Overexpression of Cx26 promoted tumor growth, EMT (reduced E-cadherin; elevated Vimentin and Slug), migration, and invasion in part via the PI3K/Akt pathway [67]. Conversely, knockdown of Cx26 reversed EMT, leading to reduced migration and invasion in gefitinib-resistant sublines of HCC827 and PC9 cells [67]. Thus, this study suggested a promoting role of cytoplasmic Cx26 in tumor growth, EMT and cancer cell invasion via activating PI3K/Akt pathway (Fig. 3).

### Increased expression and membrane localization of connexin 26 in metastatic lesions

On the other hand, similar to Cx43, increased expression and membrane-localization of Cx26 was found in metastatic lesions [18, 64]. In colorectal cancer, elevated Cx26 levels were found in lung metastatic lesions in comparison with their paired primary tumors [64]. In breast cancer, the expression of Cx26 was increased in metastatic lymph nodes as compared to paired primary tumors [18]. Importantly, the cell surface Cx26 was only found in metastatic lesions of breast cancer [18]. However, the clinical correlation of cell surface Cx26 with metastasis and prognosis needs further study (see Table 2).

### Connexin 26-mediated GJIC promotes migration as single cells via reducing cell-cell adhesion

As previously described, cell surface connexins are capable of forming gap junctions. The promoting role of Cx26-GJIC in metastasis was first described in the study by Akihiko et al. In this study, Cx26 was found to be overexpressed in the metastatic subline (BL6) of B16 mouse melanoma cells, as compared to the non-metastatic subline (F10) [68]. Ectopic expression of Cx26 enhanced the metastasis of F10 cells [68]. On the other hand, expression of the Cx26-C60F mutant, which caused a dominant-negative effect on Cx26-mediated GJIC [69], inhibited the metastasis of BL6 cells [68]. Thus, Cx26-mediated GJIC was suggested to promote metastasis (Fig. 3).

Furthermore, a study of cervical cancer revealed the promoting role of Cx26-GJIC in cancer cell migration. In HeLa cells, overexpression of wild-type Cx26 led to enhanced GJIC and a unique migration pattern, which was not observed in control cells or cells expressing membrane-localized yet GJIC-defective mutants (R75Y and T135A of Cx26); wild-type Cx26 overexpressed HeLa cells tended to separate from one another and migrated as single cells [22]. Thus, only the wild-type but not mutant Cx26 enhanced the migration of HeLa cells [22]. Moreover, HeLa cells overexpressing Cx26 showed decreased N-cadherin, which was suggested as the major adhesion molecule for the HeLa cells [22]. Overexpression of N-cadherin in HeLa cells partially reversed the migration activity enhanced by Cx26 overexpression [22]. Thus, Cx26-GJIC seemed to promote migration as single cells via reducing cell-cell adhesion (Fig. 3).

It seemed paradoxical that Cx26-GJIC can promote cell migration, a process known to involve the disruption of GJIC [22]. Polusani et al. speculated that Cx26-GJIC might be involved in transient contact of cancer cells with surrounding tissues, leading to enhanced cell migration as single cells by reducing N-cadherin [22], yet further studies are needed to resolve this paradox. The Cx26-GJIC seems to participate in a special function of metastatic cancer cells. In contrast, cytoplasmic Cx26 may promote tumor growth, EMT, migration, and invasion via PI3K/Akt pathway [67], suggesting the broad involvement of cytoplasmic Cx26 in multiple functions of tumor progression.

## Connexin 32

### Cytoplasmic connexin 32 promotes proliferation, migration and metastasis

Similar to cytoplasmic Cx26, cytoplasmic Connexin 32 (Cx32, GJB1) was found to play promoting roles in metastasis. Cx32 expressed in breast tumors was found to be localized in the cytoplasm, and increased levels of Cx32 were observed in metastatic lymph nodes versus primary tumors [70]. Cytoplasmic Cx32 was also found in primary liver tumors [71]. In Huh7 hepatoma cells, overexpressed Cx32 was found to be exclusively localized in the cytoplasm [72]. Overexpression of Cx32 in Huh7 cells promoted the proliferation, migration, invasion in vitro, and metastasis in an intrahepatic mouse model [72] (Fig. 3). However, how cytoplasmic Cx32 promoted metastatic behaviors of Huh7 cells remains to be elucidated.

### Connexin 32-mediated GJIC inhibits migration via enhancing cell-cell aggregation

Unlike Cx26-GJIC, Cx32-GJIC was reported to inhibit cancer migration. In HeLa cervical cancer cells, overexpression of Cx32 increased GJIC and cell-cell aggregation, leading to decreased migration and invasion [73]. Treatment of GJIC inhibitor oleamide partially reversed the cell aggregation enhanced by Cx32, resulting in increased cell migration and invasion [73]. In the same study, they also found that Cx32 independent of GJIC could promote cell-cell aggregation via activation of p38 and ERK1/2 [73] (Fig. 3).

The expression of Cx32 was reported to play suppressive roles in Caki-1 renal carcinoma (RCC) and SMMC-7721 hepatocellular carcinoma (HCC) cells, where overexpression of Cx32 led to increased GJIC [74, 75]. In Caki-1 cells, overexpression of Cx32 decreased in vitro cell migration and in vivo metastasis in a tail vein injection model [76]. Cx32 overexpression in Caki-1 cells reduced HIF1α, HIF2α, fibrinolytic factor PAI-1 and Src activation, which were enhanced under hypoxic incubation [76, 77]. In SMMC-7721 cells, knockdown of Cx32 enhanced EMT (reduced E-cadherin; elevated Vimentin and Snail), migration and invasion [78]. In another study, overexpression of Cx32 in SMMC-7721 cells enhanced the acetylation of p53, leading to prolonged half-life of p53 [79]. Knockdown of p53 rescued the migration inhibited by Cx32 overexpression, suggesting that Cx32 inhibited migration of SMMC-7721 cells via p53 [79]. However, in the above studies, the Cx32 subcellular localization was not characterized.

## Connexin 31, 31.1, 46, and 30.3

As Cx43, Cx26, and Cx32 are the most studied connexins in cancer, the roles of other isotypes of connexin in metastasis were less known. In H1299 NSCLC cells, overexpressed of Connexin 31.1 (Cx31.1, GJB5) was found to be localized in the endoplasmic reticulum (ER) and lysosome [80]. Overexpression of Cx31.1 in H1299 cells inhibited migration and invasion, which were rescued by knockdown of Cx31.1 [80]. Inhibited EMT (decreased Vimentin and increased Cytokeratin 18) was also found in H1299 cells overexpressing Cx31.1 [80]. The expression level of Connexin 31 (Cx31, GJB3) was found to be decreased in primary tumors of papillary thyroid cancer [81]. Ectopic expression of Cx31 reduced the migration and invasion of IHH-4 and BCPAP papillary thyroid cancer cells [81]. The above studies suggested the suppressive roles of cytoplasmic Cx31.1 in migration and invasion, while the subcellular localization of Cx31 in papillary thyroid cancer cells remains to be characterized. In addition, Connexin 46 (Cx46, GJA3) was found to be overexpressed in highly-invasive 95D than 95C lung cancer cells [82]. In the same study, they further found that Cx46 was targeted by miR-610, which inhibited the invasion of 95D cells [82]. This study thus suggested the promoting role of Cx46 in cancer invasion, while its subcellular localization remains to be characterized.

Recently, we found that Connexin 30.3 (Cx30.3, GJB4) promoted tumor growth, stemness and metastasis of lung cancer cells [83]. Cx30.3 was found to be induced by IGF-1 and promoted Src activation via receptor tyrosine kinase MET in C10F4 and H1650 lung cancer cells, leading to enhanced metastasis, chemoresistance, sphere formation and anchorage-independent growth [83]. We also found that Cx30.3 had no GJIC function, thus its oncogenesis promoting functions must be resulted from other Cx30.3-mediated signaling pathways, however, the precise subcellular localization of C30.3 remains to be characterized [83].

## Connexins in tumor stroma

### Increased connexin 43, 26, and 30 levels in tumor stroma

Although the role of connexins expressed in cancer cells are well recognized, alteration of stromal connexin levels and their correlation with metastasis is less studied, yet it could be important as well. Higher Cx43 levels were found in stromal tissues of tumors from patients with metastasis (M1) than from patients without (M0) in colon cancer, suggesting stromal Cx43 may act as a potential marker for metastasis in colorectal adenocarcinoma [84]. Similarly, higher Cx43 levels were found in bone marrow stromal cells (BMSCs) derived from multiple myeloma patients than from healthy donors [85]. The expression of Cx26 and Cx30 (GJB6) was elevated in the epidermal layers adjacent to tumors from melanoma patients and was correlated with metastasis [86]. These studies suggested the potential involvement of stromal connexins in cancer progression and metastasis.

### Stromal Cx43 promotes migration and invasion via GJIC

A number of studies further revealed the functional roles of connexins expressed in stromal cells. Bone marrow stromal cells directly co-cultured with RPMI-8226 multiple myeloma cells showed increased Cx43 expression and GJIC [85]. Enhanced migration was observed in RPMI-8226 and XG-7 multiple myeloma (MM) cells when co-cultured with BMSCs [85]. Treatment with gap junction inhibitor 18-α-glycyrrhetinic acid (18αGA) reduced multiple myeloma cell adhesion to BMSCs, and partially abrogated their migration ability enhanced by co-culturing with BMSCs [85]. In another study, decreased dissemination of GL261 glioma cells into the brain parenchyma was observed in Cx43-null mice [87]. Moreover, replacement of the wild-type Cx43 with the GJIC-deficient mutant (K258stop) in astrocytes inhibited the percentage of infiltrative glioma tumor edge [87]. Overall, stromal Cx43, consistent with the Cx43 in cancer cells described above, seems to promote metastasis via GJIC.

## Effects of connexins on chemotherapy

### Bystander effects mediated by GJIC

As described in the Background section, GJIC allows the exchange of small molecules (< 1 kDa) between cells. As a result, antitumor compounds and toxic metabolites were found to diffuse to neighboring cells via GJIC, increasing cancer cell death [10]. This phenomenon has been called the bystander effect [8]. Ganciclovir (GCV) was known to be converted into a toxic metabolite by thymidine kinase (TK) and thus eliminating TK-positive (TK^+^) cells [88]. In a co-culture system of TK^+^ and TK^−^ glioma cells, overexpression of Cx43 enhanced the bystander effect of TK^−^ cells killing under GCV treatment [8]. Treatment with gap junction inhibitor 18αGA abolished the bystander effect enhanced by Cx43 overexpression [8]. Besides Cx43, Cx26 and Cx32 can also increase the bystander effect of C6 glioma cells under TK/GCV treatment [89]. A similar phenomenon was also observed in cells of esophageal squamous cell carcinoma (ESCC) [90].

Other than TK/GCV treatment, bystander effects mediated by GJIC were also observed for multiple anticancer drugs. Ectopic expression of Cx32 enhanced the killing effect of vinblastine, a tubulin inhibitor and an anticancer drug, for renal cell carcinoma (RCC) [91, 92]. Treatment with gap junction inhibitor 18αGA partially abrogated the vinblastine-induced cytotoxicity in Caki-1 RCC cells, suggesting Cx32 mediated bystander effects through GJIC [93]. Similar results were reported in another study of lung cancer, where Cx32 was shown to enhance the cytotoxicity of vinorelbine [94], another tubulin inhibitor and a chemotherapeutic for lung cancer [95]. The cytotoxicity effect by Cx32 was partially abrogated by treatment with gap junction inhibitor 18αGA [94]. In pancreatic cancer cells, overexpression of Cx26 led to increased GJIC and thus enhanced the bystander effects of gemcitabine treatment [10].

### The GJIC-independent role of connexins in chemotherapy

Other than GJIC-mediated bystander effects, some studies reported GJIC-independent roles of connexins in the efficacy of chemotherapeutics via regulating apoptosis or PI3K/Akt pathways. In U251 and T98G glioma cells, overexpressed Cx43 was predominantly localized in the cytoplasm and nucleus [96]. Overexpression of Cx43 reduced the levels of anti-apoptotic Bcl-2, and thus enhanced paclitaxel- or etoposide-induced apoptosis of U251 and T98G glioma cells [32]. This etoposide-induced apoptosis was not significantly affected by inhibition of GJIC, suggesting a GJIC-independent role of Cx43 in promoting etoposide-induced apoptosis [32]. A similar study also showed that Cx43 interacted with pro-apoptotic Bax. Overexpression of Cx43 enhanced the cleaved (active) form of Bax in sunitinib-treated H28 mesothelioma cells in a GJIC-independent manner [97]. The gefitinib-resistant sublines of HCC827 and PC9 lung adenocarcinoma cells exhibited increased Cx26 expression but not GJIC [67]. Overexpression of Cx26 in HCC827 and PC9 cells increased the phosphorylation of Akt at Ser473 and gefitinib resistance, which was reversed by further treatment with PI3K inhibitor LY294002 [67]. Therefore, connexins may play a GJIC-independent role in affecting the efficacy of chemotherapeutics.

## Factors contributing to the variety of connexin-mediated functions

For a connexin isotype, it may function differently, sometimes even oppositely, to affect metastasis and chemoresistance depending on its localization on the plasma membrane or in the cytoplasm. An important question is how the localization of connexins is regulated during tumor progression. As the most studied connexin, Cx43 localization was found to be regulated by its phosphorylation [98]. As summarized in another review, multiple Cx43 bands were observed in the SDS-PAGE of cell lysates and were named according to their phosphorylation levels, including P0, P1, P2 and P3 [99]. The phosphorylation and localization of Cx43 were found to be dynamically changing during cell cycle [99]. At G0 stage, Cx43 was found predominantly in cell surface and immunoblotted mainly as P2 isoform [99]. In contrast, Cx43 was localized in the cytoplasm during mitosis with the appearance of P3 isoform in immunoblotting [99]. Cx43 in colon tumor cells was found to be in the P0 form, while both P0-Cx43 and P2-Cx43 were found in normal mucosa cells [21]. In addition, some studies reported that microtubule polymerization was required for the trafficking of Cx43 from the cytoplasm to the cell surface [100, 101]. Treatment with microtubule polymerization inhibitor colchicine or nocodazole reduced the membrane trafficking of Cx43 [100, 101]. Similarly, treatment with nocodazole attenuated the dye transfer of HeLa cells overexpressing Cx26 [102]. Thus, connexin phosphorylation and microtubule polymerization could be altered during carcinogenesis and metastasis, leading to altered cytoplasmic localization of connexin in primary tumor cells and increased membrane localization of connexins in metastatic lesions.

Another question is how distinct connexin isotypes function differently. For instance, cytoplasmic Cx43 was found to inhibit proliferation [21, 33], while the cytoplasmic Cx26 was shown to promote cell proliferation [67]. As the sequences of C-terminal tails of various connexin isotypes have low similarity [5], they could interact with different signaling mediators [103], leading to various downstream effects. Cx43 was found to reduce the levels of anti-apoptotic Bcl-2, leading to enhanced cell apoptosis [32]. In contrast, cytoplasmic Cx26 was found to activate PI3K/Akt pathway, leading to enhanced tumor formation, EMT, migration, and invasion [67].

As another example, Cx26-GJIC was found to facilitate cell detachment by reducing N-cadherin [22], while Cx32-GJIC increased cell-cell aggregation [73]. Similarly, as the sequences of C-terminal tails of various connexin isotypes showed low similarity, connexin isotypes may interact with different proteins on the cell surface [103]. Cx32 was found to interact with tight junction proteins (occludin and claudin), and Cx32 overexpression was found to enhance tight junction [104, 105], which was known to inhibit tumor progression and metastasis [106]. Thus, it is possible that different adhesion or junctional proteins were regulated by gap junctions of various connexin isotypes, leading to different impacts on cell-cell adhesion. Thus, for the study of a connexin isotype, its subcellular localization and interacting partners in selected cells may be critical in determining its functional roles.

## Therapeutic applications of connexins as targets

### Compounds against connexins as potential anti-metastatic drugs

The role of connexins in metastatic behaviors and sensitivity to chemotherapeutics of cancer cells has been intensively studied and has revealed therapeutic potentials of using connexins as targets to develop drugs against metastasis and chemoresistance. Metastasis inhibitor-18 (MI-18), a derivative of oleamide inhibiting Cx26-GJIC, was able to suppress metastasis of BL6 mouse melanoma cells [107]. Distilled fractions of camellia oil were found to suppress Cx26-mediated GJIC and spontaneous metastasis of BL6 cells to the lung [108]. In a rat C6 glioma model, treatment with a monoclonal antibody targeting the second extracellular loop of Cx43 (MAbE2Cx43) in combination with or without radiotherapy led to decreased tumor burden and increased survival [53]. In another study, Silvia Ferrati and her colleagues developed a novel connexin-based material with migration inhibitory activity [109]. In their study, the plasma membrane of HeLa or MDA-MB-231 cells overexpressing Cx43-YFP were harvested and processed into vesicles of similar size by cell blebbing [109, 110], and thus obtained connexin-rich membrane vesicles were named GJ vesicles [109]. Treatment with those GJ vesicles significantly reduced the migration of MDA-MB-231 cells [109] (Table 3).

**Table 3:** Novel compounds that target connexins to inhibit metastasis

Name	Cancer Type	Description	Ref.
Metastasis inhibitor-18 (MI-18)	Melanoma	Inhibited Cx26-mediated GJIC and metastasis.	[107]
Distilled fraction of camellia oil	Melanoma	Inhibited Cx26-mediated GJIC and metastasis.	[108]
MAbE2Cx43	Glioma	A monoclonal antibody targeting the second extracellular loop of Cx43. Decreased tumor burden and increase survival.	[53]
GJ vesicle	Breast	Connexin-rich membrane vesicle derived from Cx43-overexpressing HeLa or MDA-MB-231 cells. Decreased cell migration.	[109]
αCT-1	Breast	A 25 amino acid peptide drug that mimics a cytoplasmic region of Cx43. Enhanced Cx43-GJIC, leading to improved efficacy of tamoxifen and lapatinib.	[112, 113, 115]
TAT-Cx43_266-283_	Glioma	A cell-penetrating peptide based on Cx43 (amino acids 266–283). Inhibited cell migration and invasion.	[118]
PQ1	Breast	Increased Cx43 and decreased Cx46 expression.	[121]
4-OH-tamoxifen	Breast	Decreased Cx43 expression and migration.	[122]
Fulvestrant	Breast	Decreased Cx43 expression and migration.	[122]
Ginsenoside	PTC	Increased Cx31 expression, leading to decreased cell proliferation.	[81]

*GJIC* Gap junctional intercellular communication, *PTC* Papillary thyroid cancer

As described earlier in the Background section, the sequences of the extracellular loops among various connexin isotypes are highly similar [111]. Thus, specificity is a primary concern when targeting the extracellular loops of individual connexins. Instead, drugs against the cytoplasmic regions of connexins, which are highly variable among isotypes of connexin [111], were developed. The αCT-1, a 25 amino acid peptide drug that mimics a cytoplasmic region of Cx43, entered cells efficiently and bound to the PDZ2 domain of ZO-1 [112], which has been shown to reduce the size of gap junctional plaque [113]. Treatment with αCT-1 competitively inhibited the interaction between endogenous Cx43 and ZO-1, leading to increased Cx43-mediated GJIC [113]. Additionally, αCT-1 has been used to complete a phase II trial for chronic venous leg ulcers; treatment with αCT-1 increased rates of ulcer closure without treatment-related adverse events [114]. Treatment with αCT-1 in MCF7 breast cancer cells enhanced the cytotoxic effects of tamoxifen [115], a nonsteroidal anti-estrogen for breast cancer treatment [116]. Similar results were observed in BT474 breast cancer cells in that αCT-1 treatment enhanced the effectiveness of lapatinib [115], an anti-cancer drug for breast cancer [117]. A fusion polypeptide consisting of the cell-penetrating peptide fused to a different region of the cytoplasmic tail of Cx43 (Cx43_266–283_) was also reported to inhibit activation of Src and FAK via PTEN, leading to decreased migration and invasion of glioma cells [118] (Table 3).

As mentioned above, an important factor determining the functional roles of connexins is their subcellular localization, which is regulated, in part, by microtubule polymerization [100, 101]. Calder et al. developed a cell penetrating peptide named JM2, which contains the microtubule binding domain of Cx43 [119]. Treatment with JM2 inhibited the membrane trafficking of Cx43 in human umbilical vein endothelial cells (HUVECs) [120] and body inflammation surrounding silicone implants [119]. The authors also mentioned the potential usage of JM2 in cancer treatment [120].

### Connexins involved in the anti-metastatic effects of therapeutics

Connexins were also reported to be involved in the anti-metastatic effects of different chemotherapeutic drugs. A recent study found that a substituted quinoline, PQ1, increased Cx43 levels at early stages of tumor formation and decreased Cx46 levels at late stages of tumor formation [121]. Inhibition of estrogen receptor by fulvestrant or 4-OH-tamoxifen reduced Cx43 levels and migration of ER-positive MCF-7 and BT474 breast cancer cells [122]. Ginsenoside (Rg1) induced the expression of Cx31, resulting in the decreased proliferation of papillary thyroid cancer cells [81] (Table 3). These results suggested that connexins may be up- or down-regulated in the presence of certain chemotherapeutics. Based on the promoting or inhibitory role of different connexins described above, those reagents could potentially be exploited for connexin-mediated cancer treatment.

### Concerns of targeting connexins and potential strategies

In consideration of the multiple roles of cell surface connexins, targeting cell surface connexins is especially challenging to serve as an anti-tumor modality. For primary tumor cells expressing cell surface Cx43, targeting Cx43 may inhibit their metastatic abilities yet promote their proliferation. Conversely, forced expression of cell surface Cx43 in cancer cells may inhibit tumor growth yet promote their metastatic potential. On the other hand, targeting cell surface Cx43 may be considered in patients where primary tumor cells expressing only cytoplasmic Cx43, since it has been shown that Cx43 is re-expressed on cell surface in metastatic tumors [18]. Thus, targeting cell surface Cx43 may be considered as an anti-metastasis strategy for those tumors expressing only cytoplasmic Cx43. In addition, considering that Cx43-GJIC may facilitate the transmission of chemotherapeutics to facilitate bystander effects of the drug, targeting cell surface Cx43 should not be implemented in combination with treatment of chemotherapeutics.

## Conclusions

Studies over the past two decades have suggested that connexins, especially Cx43, Cx26, and Cx32, can serve as biomarkers for prognosis of metastasis and survival, as well as acting as essential players in metastasis and chemoresistance. Importantly, one connexin isotype may contribute differently, sometimes even oppositely, to tumor progression and chemoresistance. Thus, a precise strategy is needed for each patient to avoid unwanted side-effects. Moreover, GJIC mediated by different connexin isotypes, such as Cx26 and Cx32, may contribute oppositely to tumor progression. Thus, connexin isotype-specific GJIC inhibitors instead of pan-GJIC inhibitors should preferably be developed. In addition, except Cx43, Cx26, and Cx32, other connexins were much less studied. Further investigation of their functional roles in cancer could lead to the identification of novel biomarkers and targets for prognosis and therapeutic development against metastasis and chemoresistance.

## Abbreviations

18αGA: 18-α-glycyrrhetinic acid
AT: Amino-terminus
BMSC: Bone marrow stromal cell
CBX: Carbenoxolone
CT: C-terminal tail
Cx26: Connexin 26 or GJB2
Cx30: Connexin 30 or GJB6
Cx30.3: Connexin 30.3 or GJB4
Cx31: Connexin 31 or GJB3
Cx31.1: Connexin 31.1 or GJB5
Cx32: Connexin 32 or GJB1
Cx43: Connexin 43 or GJA1
Cx46: Connexin 46 or GJA3
EL: Extracellular loop
EMT: Epithelial-mesenchymal transition
ESCC: Esophageal squamous cell carcinoma
GCV: Ganciclovir
GJIC: Gap junctional intercellular communication
MI-18: Metastasis inhibitor-18
NSCLC: Non-small cell lung cancer
OSCC: Oral squamous cell carcinoma
RCC: Renal cell carcinoma
TK: Thymidine kinase

## References

[1] Fitzmaurice C, Allen C, Barber RM, Barregard L, Bhutta ZA, Global Burden of Disease Cancer Collaboration (2017). Global, regional, and national cancer incidence, mortality, years of life lost, years lived with disability, and disability-adjusted life-years for 32 cancer groups, 1990 to 2015: a systematic analysis for the global burden of disease study. JAMA Oncol.

[2] Duggan MA, Anderson WF, Altekruse S, Penberthy L, Sherman ME (2016). The surveillance, epidemiology, and end results (SEER) program and pathology: toward strengthening the critical relationship. Am J Surg Pathol.

[3] Riggi N, Aguet M, Stamenkovic I (2018). Cancer metastasis: a reappraisal of its underlying mechanisms and their relevance to treatment. Annu Rev Pathol.

[4] Valastyan S, Weinberg RA (2011). Tumor metastasis: molecular insights and evolving paradigms. Cell.

[5] Laird DW (2006). Life cycle of connexins in health and disease. Biochem J.

[6] Esseltine JL, Laird DW. Next-generation connexin and pannexin cell biology. Trends Cell Biol. 2016; Available from: doi: 10.1016/j.tcb.2016.06.003 10.1016/j.tcb.2016.06.003 27339936

[7] Boitano S, Dirksen ER, Evans WH (1998). Sequence-specific antibodies to connexins block intercellular calcium signaling through gap junctions. Cell Calcium.

[8] Sanson M, Marcaud V, Robin E, Valéry C, Sturtz F, Zalc B (2002). Connexin 43-mediated bystander effect in two rat glioma cell models. Cancer Gene Ther.

[9] Gupta A, Anderson H, Buo AM, Moorer MC, Ren M, Stains JP (2016). Communication of cAMP by connexin43 gap junctions regulates osteoblast signaling and gene expression. Cell Signal.

[10] Garcia-Rodríguez L, Pérez-Torras S, Carrió M, Cascante A, García-Ribas I, Mazo A (2011). Connexin-26 is a key factor mediating gemcitabine bystander effect. Mol Cancer Ther.

[11] Goodall H (1986). Maro *B. major* loss of junctional coupling during mitosis in early mouse embryos. J Cell Biol.

[12] Stein LS, Boonstra J, Burghardt RC (1992). Reduced cell-cell communication between mitotic and nonmitotic coupled cells. Exp Cell Res.

[13] Vinken M, Vanhaecke T, Papeleu P, Snykers S, Henkens T, Rogiers V (2006). Connexins and their channels in cell growth and cell death. Cell Signal.

[14] Coller HA (2014). Is cancer a metabolic disease?. Am J Pathol.

[15] Aasen T, Mesnil M, Naus CC, Lampe PD, Laird DW (2016). Gap junctions and cancer: communicating for 50 years. Nat Rev Cancer.

[16] Banerjee D (2016). Connexin’s connection in breast cancer growth and progression. Int J Cell Biol.

[17] Phillips SL, Williams CB, Zambrano JN, Williams CJ, Yeh ES (2017). Connexin 43 in the development and progression of breast cancer: what’s the connection? (Review). Int J Oncol.

[18] Kanczuga-Koda L, Sulkowski S, Lenczewski A, Koda M, Wincewicz A, Baltaziak M (2006). Increased expression of connexins 26 and 43 in lymph node metastases of breast cancer. J Clin Pathol.

[19] Tang B, Peng Z-H, Yu P-W, Yu G, Qian F (2011). Expression and significance of Cx43 and E-cadherin in gastric cancer and metastatic lymph nodes. Med Oncol.

[20] Zhang YW, Morita I, Ikeda M, Ma KW, Murota S (2001). Connexin43 suppresses proliferation of osteosarcoma U2OS cells through post-transcriptional regulation of p27. Oncogene.

[21] Sirnes S, Bruun J, Kolberg M, Kjenseth A, Lind GE, Svindland A (2012). Connexin43 acts as a colorectal cancer tumor suppressor and predicts disease outcome. Int J Cancer.

[22] Polusani SR, Kalmykov EA, Chandrasekhar A, Zucker SN, Nicholson BJ (2016). Cell coupling mediated by connexin 26 selectively contributes to reduced adhesivity and increased migration. J Cell Sci.

[23] Elzarrad MK, Haroon A, Willecke K, Dobrowolski R, Gillespie MN, Al-Mehdi A-B (2008). Connexin-43 upregulation in micrometastases and tumor vasculature and its role in tumor cell attachment to pulmonary endothelium. BMC Med.

[24] Stoletov K, Strnadel J, Zardouzian E, Momiyama M, Park FD, Kelber JA (2013). Role of connexins in metastatic breast cancer and melanoma brain colonization. J Cell Sci.

[25] Lamiche C, Clarhaut J, Strale P-O, Crespin S, Pedretti N, Bernard F-X (2012). The gap junction protein Cx43 is involved in the bone-targeted metastatic behaviour of human prostate cancer cells. Clin Exp Metastasis.

[26] Lin JH-C, Takano T, Cotrina ML, Arcuino G, Kang J, Liu S (2002). Connexin 43 enhances the adhesivity and mediates the invasion of malignant glioma cells. J Neurosci.

[27] Zhang W, Nwagwu C, Le DM, Yong VW, Song H, Couldwell WT (2003). Increased invasive capacity of connexin43-overexpressing malignant glioma cells. J Neurosurg.

[28] Oliveira R, Christov C, Guillamo JS, de Boüard S, Palfi S, Venance L (2005). Contribution of gap junctional communication between tumor cells and astroglia to the invasion of the brain parenchyma by human glioblastomas. BMC Cell Biol.

[29] Tang B, Peng Z-H, Yu P-W, Yu G, Qian F, Zeng D-Z (2013). Aberrant expression of Cx43 is associated with the peritoneal metastasis of gastric cancer and Cx43-mediated gap junction enhances gastric cancer cell diapedesis from peritoneal mesothelium. PLoS One.

[30] Spagnol G, Trease AJ, Zheng L, Gutierrez M, Basu I, Sarmiento C, et al. Connexin43 carboxyl-terminal domain directly interacts with β-Catenin. Int J Mol Sci 2018;19. Available from: doi: 10.3390/ijms19061562 10.3390/ijms19061562 PMC6032326 29882937

[31] Ai Z, Fischer A, Spray DC, Brown AM, Fishman GI (2000). Wnt-1 regulation of connexin43 in cardiac myocytes. J Clin Invest.

[32] Huang RP, Hossain MZ, Huang R, Gano J, Fan Y, Boynton AL (2001). Connexin 43 (cx43) enhances chemotherapy-induced apoptosis in human glioblastoma cells. Int J Cancer.

[33] Xu H-T, Li Q-C, Zhang Y-X, Zhao Y, Liu Y, Yang Z-Q (2008). Connexin 43 recruits E-cadherin expression and inhibits the malignant behaviour of lung cancer cells. Folia Histochem Cytobiol.

[34] Alaga KC, Crawford M, Dagnino L, Laird DW (2017). Aberrant Cx43 expression and mislocalization in metastatic human melanomas. J Cancer.

[35] Kańczuga-Koda L, Sulkowska M, Koda M, Reszeć J, Famulski W, Baltaziak M (2003). Expression of connexin 43 in breast cancer in comparison with mammary dysplasia and the normal mammary gland. Folia Morphol.

[36] Liang Q-L, Wang B-R, Chen G-Q, Li G-H, Xu Y-Y (2010). Clinical significance of vascular endothelial growth factor and connexin43 for predicting pancreatic cancer clinicopathologic parameters. Med Oncol.

[37] Zhang Y, Xu H, Wang E (2005). Expressions of connexin 43 and E-cadherin and their correlation in non-small cell lung cancer. Zhongguo Fei Ai Za Zhi.

[38] Sun W-H, Liu H-M, Li Y-J, Ji X-R, Liang D-P (2004). A study of the relationship between the expression of connexin43, E-cadherin and biological behaviors of human laryngeal cancer. Zhonghua Er Bi Yan Hou Ke Za Zhi.

[39] Puzzo L, Caltabiano R, Parenti R, Trapasso S, Allegra E (2016). Connexin 43 (Cx43) expression in laryngeal squamous cell carcinomas: preliminary data on its possible prognostic role. Head Neck Pathol.

[40] Smyth JW, Hong T-T, Gao D, Vogan JM, Jensen BC, Fong TS (2010). Limited forward trafficking of connexin 43 reduces cell-cell coupling in stressed human and mouse myocardium. J Clin Invest.

[41] Klaunig JE, Kamendulis LM (2004). The role of oxidative stress in carcinogenesis. Annu Rev Pharmacol Toxicol.

[42] Sosa V, Moliné T, Somoza R, Paciucci R, Kondoh H, LLeonart ME (2013). Oxidative stress and cancer: an overview. Ageing Res Rev.

[43] Ming J, Zhou Y, Du J, Fan S, Pan B, Wang Y, et al. miR-381 suppresses C/EBPα-dependent Cx43 expression in breast cancer cells. Biosci Rep 2015;35. Available from: doi: 10.1042/BSR20150167 10.1042/BSR20150167 PMC4643328 26450928

[44] Lin Z-J, Ming J, Yang L, Du J-Z, Wang N, Luo H-J (2016). Mechanism of regulatory effect of MicroRNA-206 on connexin 43 in distant metastasis of breast cancer. Chin Med J.

[45] Ming J, Zhou Y, Du J, Fan S, Pan B, Wang Y, et al. Identification of miR-200a as a novel suppressor of connexin 43 in breast cancer cells. Biosci Rep 2015;35. Available from: doi: 10.1042/BSR20150153 10.1042/BSR20150153 PMC4613673 26283635

[46] Gava F, Rigal L, Mondesert O, Pesce E, Ducommun B, Lobjois V (2018). Gap junctions contribute to anchorage-independent clustering of breast cancer cells. BMC Cancer.

[47] Elias LAB, Wang DD, Kriegstein AR (2007). Gap junction adhesion is necessary for radial migration in the neocortex. Nature.

[48] el-Sabban ME, Pauli BU (1991). Cytoplasmic dye transfer between metastatic tumor cells and vascular endothelium. J Cell Biol.

[49] Zhang A, Hitomi M, Bar-Shain N, Dalimov Z, Ellis L, Velpula KK (2015). Connexin 43 expression is associated with increased malignancy in prostate cancer cell lines and functions to promote migration. Oncotarget.

[50] Behrens J, Kameritsch P, Wallner S, Pohl U, Pogoda K (2010). The carboxyl tail of Cx43 augments p38 mediated cell migration in a gap junction-independent manner. Eur J Cell Biol.

[51] Ghosh S, Kumar A, Tripathi RP, Chandna S (2014). Connexin-43 regulates p38-mediated cell migration and invasion induced selectively in tumour cells by low doses of γ-radiation in an ERK-1/2-independent manner. Carcinogenesis.

[52] Siller-Jackson AJ, Burra S, Gu S, Xia X, Bonewald LF, Sprague E (2008). Adaptation of connexin 43-hemichannel prostaglandin release to mechanical loading. J Biol Chem.

[53] Yusubalieva GM, Baklaushev VP, Gurina OI, Zorkina YA, Gubskii IL, Kobyakov GL (2014). Treatment of poorly differentiated glioma using a combination of monoclonal antibodies to extracellular connexin-43 fragment, temozolomide, and radiotherapy. Bull Exp Biol Med.

[54] Franco L, Zocchi E, Usai C, Guida L, Bruzzone S, Costa A (2001). Paracrine roles of NAD+ and cyclic ADP-ribose in increasing intracellular calcium and enhancing cell proliferation of 3T3 fibroblasts. J Biol Chem.

[55] Rhett JM, Yeh ES. The potential for connexin hemichannels to drive breast cancer progression through regulation of the inflammatory response. Int J Mol Sci 2018;19. Available from: doi: 10.3390/ijms19041043 10.3390/ijms19041043 PMC5979453 29601539

[56] Schalper KA, Carvajal-Hausdorf D, Oyarzo MP (2014). Possible role of hemichannels in cancer. Front Physiol.

[57] Fu Y, Shao Z-M, He Q-Z, Jiang B-Q, Wu Y, Zhuang Z-G (2015). Hsa-miR-206 represses the proliferation and invasion of breast cancer cells by targeting Cx43. Eur Rev Med Pharmacol Sci.

[58] Wang Z, Wang D-Z, Pipes GCT, Olson EN (2003). Myocardin is a master regulator of smooth muscle gene expression. Proc Natl Acad Sci U S A.

[59] Li H, Xiang Y, Fan L-J, Zhang X-Y, Li J-P, Yu C-X (2017). Myocardin inhibited the gap protein connexin 43 via promoted miR-206 to regulate vascular smooth muscle cell phenotypic switch. Gene.

[60] Davidson B, Abeler VM, Førsund M, Holth A, Yang Y, Kobayashi Y (2014). Gene expression signatures of primary and metastatic uterine leiomyosarcoma. Hum Pathol.

[61] Ryszawy D, Sarna M, Rak M, Szpak K, Kędracka-Krok S, Michalik M (2014). Functional links between Snail-1 and Cx43 account for the recruitment of Cx43-positive cells into the invasive front of prostate cancer. Carcinogenesis.

[62] Wang Y, Liu J, Ying X, Lin PC, Zhou BP (2016). Twist-mediated epithelial-mesenchymal transition promotes breast tumor cell invasion via inhibition of hippo pathway. Sci Rep.

[63] Naoi Y, Miyoshi Y, Taguchi T, Kim SJ, Arai T, Tamaki Y (2007). Connexin26 expression is associated with lymphatic vessel invasion and poor prognosis in human breast cancer. Breast Cancer Res Treat.

[64] Ezumi K, Yamamoto H, Murata K, Higashiyama M, Damdinsuren B, Nakamura Y (2008). Aberrant expression of connexin 26 is associated with lung metastasis of colorectal cancer. Clin Cancer Res.

[65] Naoi Y, Miyoshi Y, Taguchi T, Kim SJ, Arai T, Maruyama N (2008). Connexin26 expression is associated with aggressive phenotype in human papillary and follicular thyroid cancers. Cancer Lett.

[66] Inose T, Kato H, Kimura H, Faried A, Tanaka N, Sakai M (2009). Correlation between connexin 26 expression and poor prognosis of esophageal squamous cell carcinoma. Ann Surg Oncol.

[67] Yang J, Qin G, Luo M, Chen J, Zhang Q, Li L (2015). Reciprocal positive regulation between Cx26 and PI3K/Akt pathway confers acquired gefitinib resistance in NSCLC cells via GJIC-independent induction of EMT. Cell Death Dis.

[68] Ito A, Katoh F, Kataoka TR, Okada M, Tsubota N, Asada H (2000). A role for heterologous gap junctions between melanoma and endothelial cells in metastasis. J Clin Invest.

[69] Duflot-Dancer A, Mesnil M, Yamasaki H (1997). Dominant-negative abrogation of connexin-mediated cell growth control by mutant connexin genes. Oncogene.

[70] Kanczuga-Koda L, Sulkowska M, Koda M, Rutkowski R, Sulkowski S (2007). Increased expression of gap junction protein--connexin 32 in lymph node metastases of human ductal breast cancer. Folia Histochem Cytobiol.

[71] Krutovskikh V, Mazzoleni G, Mironov N, Omori Y, Aguelon AM, Mesnil M (1994). Altered homologous and heterologous gap-junctional intercellular communication in primary human liver tumors associated with aberrant protein localization but not gene mutation of connexin 32. Int J Cancer.

[72] Li Q, Omori Y, Nishikawa Y, Yoshioka T, Yamamoto Y, Enomoto K (2007). Cytoplasmic accumulation of connexin32 protein enhances motility and metastatic ability of human hepatoma cells in vitro and in vivo. Int J Cancer.

[73] Yang J, Liu B, Wang Q, Yuan D, Hong X, Yang Y (2011). Connexin 32 and its derived homotypic gap junctional intercellular communication inhibit the migration and invasion of transfected HeLa cells via enhancement of intercellular adhesion. Mol Med Rep.

[74] Yang Y, Qin S-K, Wu Q, Wang Z-S, Zheng R-S, Tong X-H (2014). Connexin-dependent gap junction enhancement is involved in the synergistic effect of sorafenib and all-trans retinoic acid on HCC growth inhibition. Oncol Rep.

[75] Fujimoto E, Sato H, Shirai S, Nagashima Y, Fukumoto K, Hagiwara H (2005). Connexin32 as a tumor suppressor gene in a metastatic renal cell carcinoma cell line. Oncogene.

[76] Sato H, Hagiwara H, Senba H, Fukumoto K, Nagashima Y, Yamasaki H (2008). The inhibitory effect of connexin 32 gene on metastasis in renal cell carcinoma. Mol Carcinog.

[77] Hagiwara H, Sato H, Shirai S, Kobayashi S, Fukumoto K, Ishida T (2006). Connexin 32 down-regulates the fibrinolytic factors in metastatic renal cell carcinoma cells. Life Sci.

[78] Yang Y, Zhang N, Zhu J, Hong X-T, Liu H, Ou Y-R (2017). Downregulated connexin32 promotes EMT through the Wnt/β-catenin pathway by targeting Snail expression in hepatocellular carcinoma. Int J Oncol.

[79] Zhao B, Zhao W, Wang Y, Xu Y, Xu J, Tang K (2015). Connexin32 regulates hepatoma cell metastasis and proliferation via the p53 and Akt pathways. Oncotarget.

[80] Zhang D, Chen C, Li Y, Fu X, Xie Y, Li Y (2012). Cx31.1 acts as a tumour suppressor in non-small cell lung cancer (NSCLC) cell lines through inhibition of cell proliferation and metastasis. J Cell Mol Med.

[81] Xu L, Chen S-W, Qi X-Y, Li X-X, Sun Y-B (2018). Ginsenoside improves papillary thyroid cancer cell malignancies partially through upregulating connexin 31. Kaohsiung J Med Sci.

[82] Jin J, Li C, You J, Zhang B (2014). miR-610 suppresses lung cancer cell proliferation and invasion by targeting GJA3. Zhonghua Zhong Liu Za Zhi.

[83] Lin Y-P, Wu J-I, Tseng C-W, Chen H-J, Wang L-H. Gjb4 serves as a novel biomarker for lung cancer and promotes metastasis and chemoresistance via Src activation. Oncogene 2018; Available from: doi: 10.1038/s41388-018-0471-1 10.1038/s41388-018-0471-1 30177841

[84] Radić J, Krušlin B, Šamija M, Ulamec M, Milošević M, Jazvić M (2016). Connexin 43 expression in primary colorectal carcinomas in patients with stage III and IV disease. Anticancer Res.

[85] Zhang X, Sun Y, Wang Z, Huang Z, Li B, Fu J (2015). Up-regulation of connexin-43 expression in bone marrow mesenchymal stem cells plays a crucial role in adhesion and migration of multiple myeloma cells. Leuk Lymphoma.

[86] Haass NK, Ripperger D, Wladykowski E, Dawson P, Gimotty PA, Blome C (2010). Melanoma progression exhibits a significant impact on connexin expression patterns in the epidermal tumor microenvironment. Histochem Cell Biol.

[87] Sin WC, Aftab Q, Bechberger JF, Leung JH, Chen H, Naus CC (2016). Astrocytes promote glioma invasion via the gap junction protein connexin43. Oncogene.

[88] Beck C, Cayeux S, Lupton SD, Dörken B, Blankenstein T (1995). The thymidine kinase/ganciclovir-mediated “suicide” effect is variable in different tumor cells. Hum Gene Ther.

[89] Jimenez T, Fox WP, Naus CCG, Galipeau J, Belliveau DJ (2006). Connexin over-expression differentially suppresses glioma growth and contributes to the bystander effect following HSV-thymidine kinase gene therapy. Cell Commun Adhes.

[90] Matono S, Tanaka T, Sueyoshi S, Yamana H, Fujita H, Shirouzu K (2003). Bystander effect in suicide gene therapy is directly proportional to the degree of gap junctional intercellular communication in esophageal cancer. Int J Oncol.

[91] Rtibi K, Grami D, Selmi S, Amri M, Sebai H, Marzouki L (2017). Vinblastine, an anticancer drug, causes constipation and oxidative stress as well as others disruptions in intestinal tract in rat. Toxicol Rep.

[92] De Mulder PHM, van Herpen CML, Mulders PAF (2004). Current treatment of renal cell carcinoma. Ann Oncol.

[93] Sato H, Senba H, Virgona N, Fukumoto K, Ishida T, Hagiwara H (2007). Connexin 32 potentiates vinblastine-induced cytotoxicity in renal cell carcinoma cells. Mol Carcinog.

[94] Sato H, Fukumoto K, Hada S, Hagiwara H, Fujimoto E, Negishi E (2007). Enhancing effect of connexin 32 gene on vinorelbine-induced cytotoxicity in A549 lung adenocarcinoma cells. Cancer Chemother Pharmacol.

[95] Piccirillo MC, Daniele G, Di Maio M, Bryce J, De Feo G, Del Giudice A (2010). Vinorelbine for non-small cell lung cancer. Expert Opin Drug Saf.

[96] Huang RP, Fan Y, Hossain MZ, Peng A, Zeng ZL, Boynton AL (1998). Reversion of the neoplastic phenotype of human glioblastoma cells by connexin 43 (cx43). Cancer Res.

[97] Uzu M, Sato H, Yamada R, Kashiba T, Shibata Y, Yamaura K (2015). Effect of enhanced expression of connexin 43 on sunitinib-induced cytotoxicity in mesothelioma cells. J Pharmacol Sci.

[98] Laird DW (2005). Connexin phosphorylation as a regulatory event linked to gap junction internalization and degradation. Biochim Biophys Acta.

[99] Solan JL, Lampe PD (2005). Connexin phosphorylation as a regulatory event linked to gap junction channel assembly. Biochim Biophys Acta.

[100] Guo Y, Martinez-Williams C, Rannels DE (2003). Gap junction-microtubule associations in rat alveolar epithelial cells. Am J Physiol Lung Cell Mol Physiol.

[101] Johnson RG, Meyer RA, Li X-R, Preus DM, Tan L, Grunenwald H (2002). Gap junctions assemble in the presence of cytoskeletal inhibitors, but enhanced assembly requires microtubules. Exp Cell Res.

[102] Martin PE, Blundell G, Ahmad S, Errington RJ, Evans WH (2001). Multiple pathways in the trafficking and assembly of connexin 26, 32 and 43 into gap junction intercellular communication channels. J Cell Sci.

[103] Giepmans BNG (2004). Gap junctions and connexin-interacting proteins. Cardiovasc Res.

[104] Kojima T, Sawada N, Chiba H, Kokai Y, Yamamoto M, Urban M (1999). Induction of tight junctions in human connexin 32 (hCx32)-transfected mouse hepatocytes: connexin 32 interacts with occludin. Biochem Biophys Res Commun.

[105] Kojima T, Kokai Y, Chiba H, Yamamoto M, Mochizuki Y, Sawada N (2001). Cx32 but not Cx26 is associated with tight junctions in primary cultures of rat hepatocytes. Exp Cell Res.

[106] Martin TA, Jiang WG. Loss of tight junction barrier function and its role in cancer metastasis. Biochim Biophys Acta. 2009;1788:872–91. 10.1016/j.bbamem.2008.11.005 19059202

[107] Ito A, Morita N, Miura D, Koma Y-I, Kataoka TR, Yamasaki H (2004). A derivative of oleamide potently inhibits the spontaneous metastasis of mouse melanoma BL6 cells. Carcinogenesis.

[108] Miura D, Kida Y, Nojima H (2007). Camellia oil and its distillate fractions effectively inhibit the spontaneous metastasis of mouse melanoma BL6 cells. FEBS Lett.

[109] Ferrati S, Gadok AK, Brunaugh AD, Zhao C, Heersema LA, Smyth HDC, et al. Connexin membrane materials as potent inhibitors of breast cancer cell migration. J R Soc Interface 2017;14. Available from: doi: 10.1098/rsif.2017.0313 10.1098/rsif.2017.0313 PMC5582126 28768882

[110] Charras G, Paluch E (2008). Blebs lead the way: how to migrate without lamellipodia. Nat Rev Mol Cell Biol.

[111] Martin PEM, Evans WH (2004). Incorporation of connexins into plasma membranes and gap junctions. Cardiovasc Res.

[112] Hunter AW, Barker RJ, Zhu C, Gourdie RG (2005). Zonula occludens-1 alters connexin43 gap junction size and organization by influencing channel accretion. Mol Biol Cell.

[113] Rhett JM, Jourdan J, Gourdie RG (2011). Connexin 43 connexon to gap junction transition is regulated by zonula occludens-1. Mol Biol Cell.

[114] Ghatnekar GS, Grek CL, Armstrong DG, Desai SC, Gourdie RG (2015). The effect of a connexin43-based peptide on the healing of chronic venous leg ulcers: a multicenter, randomized trial. J Invest Dermatol.

[115] Grek CL, Rhett JM, Bruce JS, Abt MA, Ghatnekar GS, Yeh ES (2015). Targeting connexin 43 with α-connexin carboxyl-terminal (ACT1) peptide enhances the activity of the targeted inhibitors, tamoxifen and lapatinib, in breast cancer: clinical implication for ACT1. BMC Cancer.

[116] Jordan VC (1992). The role of tamoxifen in the treatment and prevention of breast cancer. Curr Probl Cancer.

[117] Bilancia D, Rosati G, Dinota A, Germano D, Romano R, Manzione L (2007). Lapatinib in breast cancer. Ann Oncol.

[118] Jaraíz-Rodríguez M, Tabernero MD, González-Tablas M, Otero A, Orfao A, Medina JM (2017). A short region of connexin 43 reduces human glioma stem cell migration, invasion, and survival through Src, PTEN, and FAK. Stem Cell Rep.

[119] Calder BW, Matthew Rhett J, Bainbridge H, Fann SA, Gourdie RG, Yost MJ (2015). Inhibition of connexin 43 hemichannel-mediated ATP release attenuates early inflammation during the foreign body response. Tissue Eng Part A.

[120] Rhett JM, Calder BW, Fann SA, Bainbridge H, Gourdie RG, Yost MJ (2017). Mechanism of action of the anti-inflammatory connexin43 mimetic peptide JM2. Am J Physiol Cell Physiol.

[121] Shishido SN, Delahaye A, Beck A, Nguyen TA (2014). The anticancer effect of PQ1 in the MMTV-PyVT mouse model. Int J Cancer.

[122] Tsai C-F, Cheng Y-K, Lu D-Y, Wang S-L, Chang C-N, Chang P-C (2018). Inhibition of estrogen receptor reduces connexin 43 expression in breast cancers. Toxicol Appl Pharmacol.

